# GPER as a Receptor for Endocrine-Disrupting Chemicals (EDCs)

**DOI:** 10.3389/fendo.2020.00545

**Published:** 2020-08-19

**Authors:** Séverine Périan, Jean-Marc Vanacker

**Affiliations:** Institut de Génomique Fonctionnelle de Lyon, Université de Lyon, Université Lyon 1, CNRS UMR5242, Ecole Normale Supérieure de Lyon, Lyon, France

**Keywords:** GPER, hormone, estrogen, pathophysiology, endocrine-disrupting chemicals (EDCs)

## Abstract

Endocrine-disrupting chemicals (EDCs) are exogenous chemicals that interfere with endogenous hormonal systems at various levels, resulting in adverse health effects. EDCs belong to diverse chemical families and can accumulate in the environment, diet and body fluids, with different levels of persistence. Their action can be mediated by several receptors, including members of the nuclear receptor family, such as estrogen and androgen receptors. The G protein-coupled estrogen receptor (GPER), a seven-transmembrane domain receptor, has also attracted attention as a potential target of EDCs. This review summarizes our current knowledge concerning GPER as a mediator of EDCs' effects.

## Endocrine-Disrupting Chemicals

According to a general definition, endocrine-disrupting chemicals (EDCs) are exogenous compounds that interfere with the endogenous hormonal axes at any level (1). This includes synthesis, metabolism, transport and delivery of hormones, and also perturbation of the expression of hormone receptors as well as with the downstream signals they convey. EDCs comprise compounds that can promote or restrict a hormonal signal (acting as agonists or antagonists, respectively). Under this broad definition, EDCs include natural molecules such as the phytoestrogens (e.g., genistein, which is abundant in soy) that modulate estrogen signaling and also synthetic compounds intended for therapeutic purposes, such as the ones used as adjuvant therapy in breast cancer. Examples of the latter category include inhibitors of aromatase used to reduce the endogenous synthesis of 17β-estradiol (E2) or tamoxifen that act as an antagonist of the estrogen receptor in mammary tumors.

EDCs also comprise chemicals that are produced for various industrial purposes, being used as components of several products (plastics, paints, flame retardants, herbicides, pesticides…), that exert unintended impacts on hormonal signaling. The number and variety (in terms of chemical structure) of molecules that display suspected or validated endocrine disrupting effects increased since years (1). Furthermore, these compounds often display high levels of resistance to natural degradation leading to their accumulation in the environment as well as in body fluids [see (2–4) for examples]. Adverse effects of EDCs have been reported in domains covering all fields related to hormonal signaling, including metabolism, reproduction, induction and progression of hormone-sensitive cancers and neurodevelopment (1).

To investigate the effects of EDCs, it is essential to identify the receptors that mediate their action as well as the downstream cascades they elicit. Given that EDCs largely impact the male and female reproductive axes, it was initially suspected that their effects were largely mediated by the sex steroid receptors (5). These include the estrogen receptors (ERs) and the androgen receptor (AR), which are members of the nuclear receptor (NR) family and act as transcription factors. In line with this, several EDCs were demonstrated to modulate the activities of these NRs. However, at least in some cases, such as that of the paradigmatic EDC Bisphenol A (BPA), the affinity of these compounds for ERα appeared far lower than that of their natural ligand (6), suggesting the existence of other proteins acting as EDC receptors. Consistently, it was shown that BPA binds to ERRγ, an orphan NR which does not recognize E2, and induces its downstream activities (7, 8). As far as we are aware, the capacity of ERRγ to serve as a receptor for EDCs other than bisphenols has not been published. In contrast, an array of publications suggests that the G protein-coupled receptor (GPER) may serve as a receptor for a vast spectrum of EDCs. The purpose of this review is to (non-exhaustively) summarize what we currently know concerning the relationships between GPER and EDCs.

## GPER, An Alternative Estrogen Receptor

**Figure 1:**
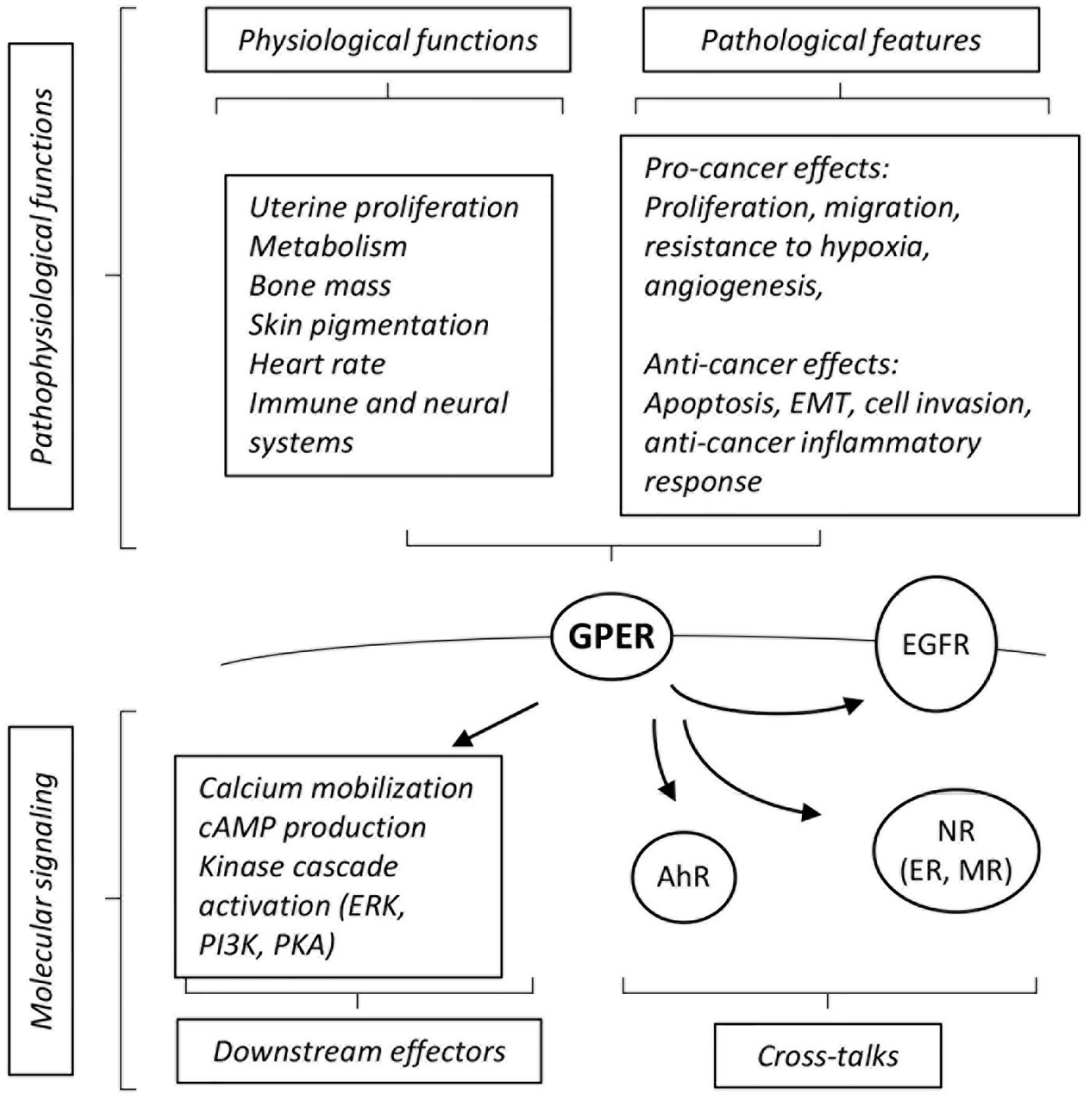
Summary of cross-talks, downstream effectors and pathophysiological effects elicited by GPER. See text for definition of abbreviations, details and references.

GPER (initially referred to as GPR30) has been identified as a membrane associated estrogen receptor 15 years ago (9, 10). This seven-transmembrane domain receptor is broadly expressed and has been detected in several sub-cellular localizations, including in internal membrane compartments, such as the endoplasmic reticulum, nucleus and even as a chromatin binding protein under certain circumstances (11). It is expected that different molecular functions could be exerted by GPER, depending on its sub-cellular localization (summarized on Figure 1). Indeed, membrane activation of GPER was shown to rapidly promote intracellular calcium mobilization, cAMP production and to induce a phosphorylation cascade in particular involving ERK1/2, PKA, and PI3K (9, 10, 12–14). On another hand, chromatin binding of GPER leads to direct transcriptional activation of target genes (11).

GPER cross-talks with different receptors to convey its downstream effects. For instance, functional interactions with the aryl-hydrocarbon receptor (AhR) or EGF receptors (EGFR) are instrumental for the activation of downstream MAPK activation (12, 15). GPER also functionally interacts with nuclear receptors at various levels. For instance, GPER is required for the effect of aldosterone mediated by the mineralocorticoid receptor (MR) in breast cancer cell lines (16). A more indirect level of cross-talks can be illustrated by the regulation of the circulating level of thyroid hormone which in turn modulates embryonic heart rate in a thyroid hormone receptor-dependent manner (17). Functional interactions between GPER and ER have been abundantly documented, may depend on the cell type considered and may lead to congruent or opposing effects [reviewed in (18)]. For example, in ovarian cancer cells, both GPER- and ER-mediated signals are involved in the activation of ERK1/2 leading to increased c-fos expression and induction of proliferation (19). On another hand, at least in ER-positive breast cancer cells, tamoxifen acts as an ER antagonist, but as a GPER agonist (9). Altogether, this shows that GPER displays a wide array of molecular functions and interactions with other signaling pathways. Given its broad expression spectrum and its described pathophysiological functions, GPER has emerged as a factor of clinical importance [reviewed in (20)].

## Pathophysiological Functions of GPER

The functions of GPER have been investigated using *in vivo* and *in vitro* approaches. GPER knocked-out mice [reviewed in (21)] reproduce normally, indicating that GPER is not absolutely required for reproduction. However, pharmacological studies (i.e., using treatments with agonists and antagonists) suggest that GPER intervenes in uterine epithelial proliferation, suggesting a subtle impact on reproductive function that may be compensated for in the absence of the receptor. Other *in vivo* studies have indicated that GPER is involved, amongst others, in glucose and lipid metabolism, bone mass, skin pigmentation, regulation of heart rate, and immune and neural systems [(17, 22–24), reviewed in (25)].

The impact of GPER, as a novel estrogen receptor, on cancers has been extensively analyzed, in particular on hormone-related cancers (e.g., breast, ovary, and endometrium). Several studies report a pro-cancer effect of GPER (26, 27). Indeed, high GPER expression correlates to a poor prognosis in breast and endometrial carcinoma (28, 29). Consistently, GPER activation promotes various traits of cancer progression including cell migration in triple negative breast cancer cells, resistance to hypoxia and proangiogenic response (30–32). GPER is also active in cancer-associated fibroblasts (CAFs) where it favors tumor-promoting activities (33, 34).

In contrast, other studies rather indicate that GPER may exert anti-cancer roles. For instance, high expression of GPER has been reported as a factor of favorable prognosis in triple-negative breast cancers (35). Similarly, low level of GPER protein expression in the cytoplasm is associated with lower levels of disease free survival in breast cancer, even when eliminating potentially confounding factors such as ER/PR/HER2 status (36). Consistently, reports indicate that GPER activation leads to cell cycle arrest, apoptosis and cell death in ER-positive and –negative cell lines (37, 38). Interestingly, an inhibitory effect of GPER has also been noted in cancers that do not depend on estrogen signaling. Indeed, GPER inhibits epithelial-to-mesenchymal transition and cell invasion in prostate and pancreatic cancer cells (39). Furthermore, tamoxifen-mediated GPER activation impairs the conversion of pancreatic stellate cells into myofibroblasts (an equivalent of CAFs in pancreatic tumors), which in turn leads to reduced cancer cell survival (40, 41). Moreover, GPER-deficient mice display increased inflammation in induced liver tumorigenesis resulting in accelerated tumor growth (42).

To date, the roles of GPER in cancer thus appear unclear. However, it is possible to propose non-mutually exclusive hypotheses to solve these apparent contradictions. (i) GPER sub-cellular localization may impact its prognosis value (and its activities). In this respect, in contrast to its detection in the cytoplasm, the low nuclear expression of GPER does not correlate to breast cancer aggressiveness (36). (ii) GPER activities may depend on the tissues in which they are studied. It may indeed be envisioned that, in pancreas and liver, the anti-inflammatory effects displayed by GPER in non-cancer cells may overcome its capacity to promote tumor growth in cancer cells. (iii) GPER may exert different activities on the various steps of cancer progression. In a mouse model of mammary cancer, GPER indeed appears dispensable for cancer initiation but contributes to the establishment of metastasis (43). (iv) GPER may play different roles depending on the expression of cross-talking factor. For example, GPER promotes the growth of ER-negative SKBr3 cells, but reduces that of ER-positive MCF7 cells (44). Furthermore, the stimulating effect of GPER on ovarian cancer cells depends on EGFR (19). More work is obviously required to refine our knowledge on the impact of GPER on cancers.

## Identifying Chemical Modulators of GPER Activities

**Figure 2:**
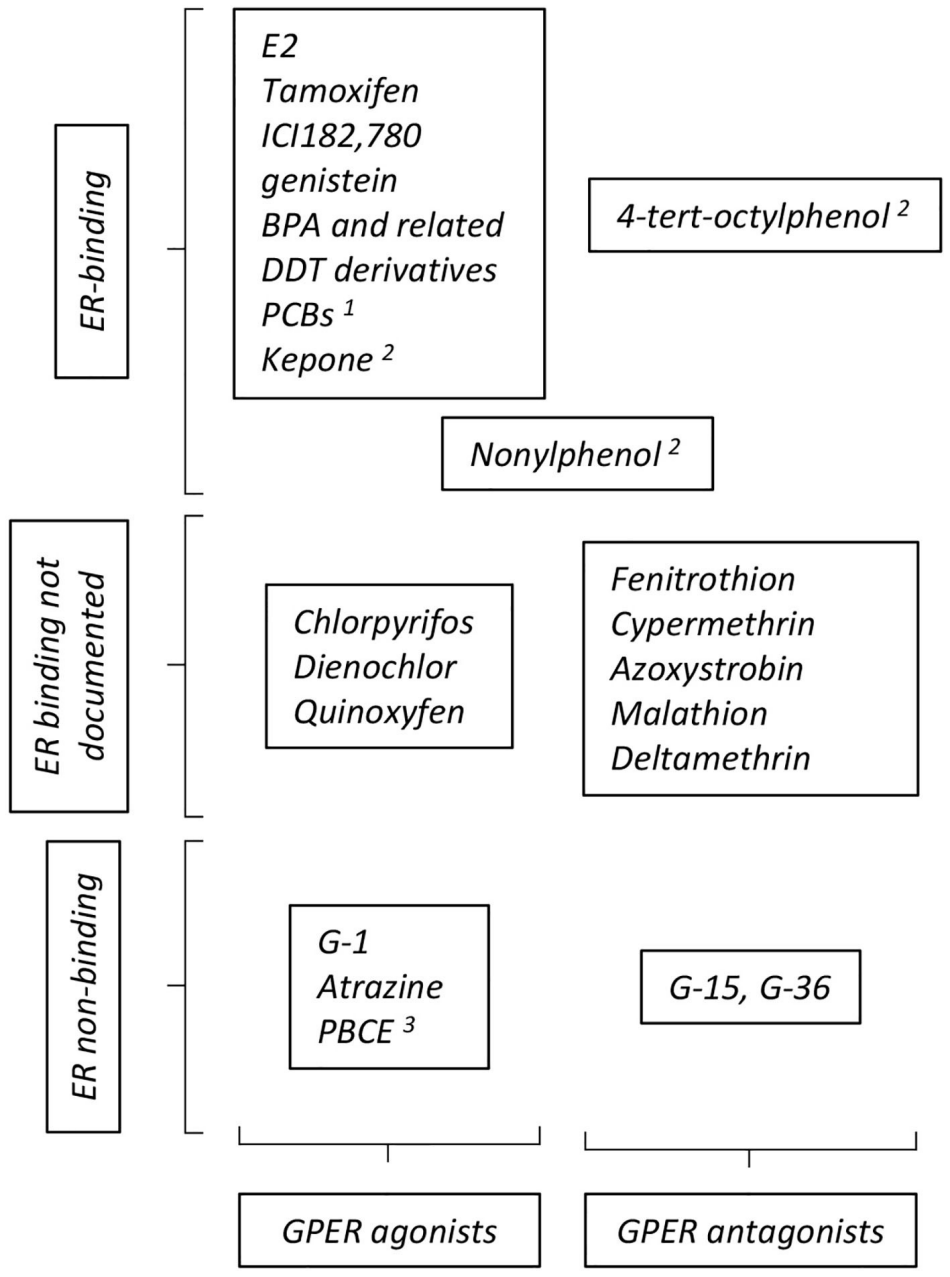
Summary of compounds reported to act as GPER- agonists and antagonists. The particular position occupied by nonylphenol reflects contradictory reports ascribing this compound as GPER- agonist or antagonist. See text for definition of abbreviations, details and reference. Additional references for ER binding: 1: (45); 2: (46); 3: (47).

GPER was identified as a functional estrogen receptor in ER-negative cells by a combination of binding and functional studies (i.e., detection of GPER-dependent calcium mobilization or adenylyl cyclase activation) (9, 10), suggesting a shared repertoire between compounds acting on ER and on GPER (summarized on Figure 2). ER-binding ligands were thus examined and this led to the surprising finding that tamoxifen and ICI182, 780 (two ER-antagonist used in adjuvant breast cancer therapy) actually acted as GPER-agonists. Furthermore, EDCs, acting as xenoestrogens on ER, including genistein, BPA, and DDT derivatives also impacted GPER, as shown by binding assays coupled to functional signaling assays (48). Although the affinity of these compounds for GPER is less than that of E2, they broadly display similar binding constants as those displayed on ER. However, the repertoires of compounds bound by GPER and ER are not strictly similar. For instance, the potent ER-agonist DES does not bind GPER (10). Moreover, functional screening identified specific synthetic GPER ligands (i.e., not recognizing the nuclear estrogen receptors) that act as agonist (G-1) or antagonists (G-15 and G-36) for GPER [reviewed in (49)]. Altogether, this shows that GPER and ER display both overlapping and distinct repertoires of compound recruitment. Furthermore, molecular modeling and *in silico* docking studies indicated that GPER offers several cavities to accommodate large volume ligands and suggested a broad number of possible binding compounds (50, 51). Indeed, competition assays and measurement of cAMP accumulation revealed that organochlorides, such as polychlorinated biphenyls (PCBs) and kepone (*aka* chlordecone), act (amongst others) as GPER agonists (48).

The GPER-dependent consequences of EDC exposure in terms of molecular outcomes, as well as at the cellular, phenotypical levels have also been studied. The pesticide atrazine does not transactivate ER but induces GPER-dependent ERK activation in ovarian cancer cells and CAF, leading to increased proliferation and migration (52).

BPA induces proliferation and migration of ER-negative breast cancer cells and CAFs in a GPER-dependent manner (53, 54). Proliferation of mouse spermatogonial and Sertoli cells has also been shown as induced by BPA through GPER (55, 56). Intriguingly, analysis of the dose-response indicated a non-monotonous effect in form of an inverted U-shaped curve. Other bisphenols, used as substitutes for BPA and found in high concentrations (similar to or higher than those of BPA) in the environment and body fluids (57) have also been tested. As compared to BPA, some of these analogs, such as BPAF and BPB, display comparable binding affinities to GPER (as determined by E2 displacement), GPER activation capacities (as assessed by calcium mobilization and cAMP production) and, GPER-dependent induction of cell migration (58). Intriguingly, BPF did not display such activities although other studies indicated that its effects on hormonal axes was comparable to those of BPA [reviewed in (59)]. Other compounds such as polybrominated diphenyl ether (PBCE, used as flame retardant additives) that, as BPA, display a diphenyl core, also display GPER binding with an affinity in the micromolar range (60). These compounds induce cAMP accumulation, calcium mobilization and cell migration in ER-negative breast cancer cells.

Nonylphenol (NP) induces cardiac contractility in a non-monotonic manner (61). The effect at low doses is antagonized by G-15, suggesting that NP acts as a GPER agonist. Such an effect of NP has also been suggested on human ER-negative cells (48) as well as on zebrafish oocyte maturation, where this compound (as well as other alkylphenols, including BPA) blocks oocyte maturation, as does G-1 (62). In contrast, NP has been shown to counteract the action of G-1 as a moderator of asthma symptoms in mouse models (63), suggesting that this compounds acts as a GPER-antagonist. Whether these apparent discrepancies originate from the differences in the pathophysiological situations that are analyzed remains to be established.

## Concluding Remarks

GPER is a promiscuous receptor displaying a broad spectrum of compound recognition, including toward EDCs. There is however a specificity of GPER-recognition within given chemical families, as exemplified by the bisphenol derivatives. It should be noted that most if not all of the studies examining the effects of EDC on GPER have been performed using cell cultures systems and seldom *in vivo*. *In vitro* cell models provide irreplaceable tools for their capacity to be experimentally manipulated. However, comparing the effects of EDCs in wild type and GPER-inactivated animals will greatly increase our understanding of the action of these compounds.

Although several of the compounds impacting on GPER have also been demonstrated to bind ERs, there is a level of selectivity, discriminating these receptors. Various levels of cross-talks have been demonstrated between GPER and other proteins such as ERs, EGFR, or AhR. Whether or not these cross-talks are effective in a given cellular system and may influence the outcome of GPER activation is not always understood. It will thus be of interest to assess the effects of EDCs as GPER modulators under conditions where these cross-talks are controlled.

GPER exerts a large array of pathophysiological functions. A level of overlap between these functions and the perturbations induced by exposure to EDCs is worth noting. Together, this places GPER as a strong candidate to mediate, at least part, of the adverse effects displayed by EDCs. As discussed above, the exact role of GPER in cancer initiation and progression is a matter of debate and may depend on the considered tissue and/or disease stage. How the modulation of GPER activities by EDCs impact cancer features is thus unclear but should be an important field of investigations in the near future.

## Author Contributions

All authors listed have made a substantial, direct and intellectual contribution to the work, and approved it for publication.

## Conflict of Interest

The authors declare that the research was conducted in the absence of any commercial or financial relationships that could be construed as a potential conflict of interest.
